# Effect of polyamide 6 on the morphology and electrical conductivity of carbon black-filled polypropylene composites

**DOI:** 10.1098/rsos.170769

**Published:** 2017-12-20

**Authors:** Xuewei Zhang, Jiang Liu, Yi Wang, Wei Wu

**Affiliations:** Sino-German Joint Research Center of Advanced Materials, School of Materials Science and Engineering, East China University of Science and Technology, Shanghai 200237, People's Republic of China

**Keywords:** immiscible blend, polyamide 6/polypropylene, carbon black, electrical conductivity, morphology, co-continuous structure

## Abstract

Carbon black (CB)-filled polypropylene (PP) with surface resistivity between 10^6^ and 10^9^ Ω sq^−1^ is the ideal antistatic plastic material in the electronics and electric industry. However, a large amount of CB may have an adverse effect on the mechanical properties and processing performance of the material, thus an improved ternary system is developed. Blends of CB-filled PP and polyamide 6 (PA6) have been prepared by melt blending in order to obtain electrically conductive polymer composites with a low electrical percolation threshold based on the concept of double percolation. The morphological developments of these composites were studied by scanning electron microscopy. The results showed that CB particles were selectively dispersed in PA6 phases due to the good interaction and interfacial adhesion between CB and PA6. At the same CB loadings, the surface resistivity of PP/PA6/CB composite was smaller than that of PP/CB composite system, which indicated the better conductivity in the former composite. The increasing amount of PA6 in the composites changed the morphology from a typical sea–island morphology to a co-continuous morphology. What is more, with 8 wt% of CB and PP/PA6 phase ratio of 70/30 in which the PP and PA6 phases formed a co-continuous structure, the electrical conductivity of the composite peaked at 2.01 × 10^5^ Ω sq^−1^.

## 1. Introduction

Electrically conductive polymer composites (CPCs) are important in technological applications and a topic of tremendous commercial interest. The CPCs are typically a polymeric matrix with conductive fillers. These multifunctional materials are employed in various commercial applications due to their advantages, such as good electrical conductivity, corrosion resistance, light weight and enhanced mechanical properties. CPCs can be used in electromagnetic interference shielding [1], electrostatic dissipation [2], heat dissipation films [3], chemical sensors [4], actuators [5] and photovoltaic devices [6].

With the addition of conductive filler, a drastic transition from electric insulator to conductor can be clearly observed when the filler content exceeds a certain critical value. This transition-like change in conductivity is generally attributed to percolation phenomena [7], and the critical filler content is known as the percolation threshold, corresponding to the formation of a conductive filler network in the polymer matrix.

Carbon nanofillers, such as carbon black (CB), carbon nanotubes and graphene, are electrically conductive and good for improving polymer properties. CB-filled polymers are widely used in industrial applications for the overwhelming cost advantage. In general, single-polymer systems require a substantial concentration of the conductive filler to achieve significant electrical conductivity in the range of 10^−9^ to 10^−3^ S cm^−1^; meanwhile the addition increases the melt viscosity and decreases the mechanical and rheological properties of the material [8]. Furthermore, high CB content will lead to local differences in resistivity, exfoliation of CB particles and so on. Therefore, how to reduce the amount of CB (percolation threshold) has gradually become a hot topic in recent years.

Several studies found that the percolation threshold was reduced using multiphase polymer systems where the conductive filler was incorporated into immiscible polymer blends [9–11]. The performance and conductivity of an electrical CPC system are determined not only by the properties but also by the morphology [12,13]. When the CB is preferentially distributed in one continuous phase or located at the interface of the two-phase matrix forming the co-continuous phase, the percolation threshold is significantly reduced. The concept of double percolation was first put forward by Sumita *et al*. [14]. The distribution of CB in the two-phase matrix was governed by the polarity (surface tension), viscosity and mixing order of the polymer matrix.

CB-filled CPCs are typically prepared using melt mixing or solution processing. Wu *et al*. [15] investigated CB-filled acrylonitrile–butadiene–styrene/polyamide 6 (PA6) blends. The CB particles were found to be preferentially localized in the PA6 phase, and with an increase in CB loading, the critical volume fraction of PA6 for building the co-continuous structure decreased. Yang *et al*. [16] investigated the electrical conductivity and impact strength of polypropylene (PP)/ethylene–propylene–diene monomer (EPDM)/CB ternary composites. The CB accumulated in the EPDM phase to form a filler-network structure, which decreased the percolation threshold with a maximum conductivity at 3 wt% CB. Shen *et al*. [17] brought CB to poly(methyl methacrylate)/PP (50/50) interface by pre-compounding CB with ethylene–acrylic acid copolymer, and the percolation threshold was only one-fifth of that in PP. Sun *et al*. [18] and Garmabi & Naficy [19] prepared a PP/PA6/CB composite and found that the CB particles adhered to the PA phase and formed conducting pathways. In addition, Dai *et al*. [20] attempted to direct the CB particles to migrate toward the interfaces between the polar and nonpolar polymeric phases using a multistage blending process and obtained a lower percolation threshold. These results indicate that the addition of a polar phase might play a crucial role in the distribution of CB particles and the formation of a conducting network.

PP is a commodity polymer, which is used in several applications because of its low density, small water absorption, chemical resistance and good mechanical performances. Owing to the lack of effective contacts between CB particles at lower loadings, turning PP into a conductive polymer requires relatively high amounts of conductive fillers such as CB. However, this may lead to a significant decrease of mechanical properties. Blending PP with an engineering polymer, such as PA6, could reduce its electrical percolation threshold (by double percolation) and bring excellent comprehensive performance and economic advantages. To our knowledge, there are few reports about the specific influence of the ratio of PA6 and PA6/PP on the microstructure and electrical property of polymer blends at the same CB loading. In this work, we attempted to develop a CB-loaded CPC. The composite is based on PA6 (a polar polymer) as dispersed phase and PP (a nonpolar polymer) as matrix. Effort was made to figure out the relations between the microstructures and the resulting properties, such as rheological and electrical properties. Microstructure of the nanocomposites was characterized by the blend morphologies and dispersion of the CB.

## 2. Experiment

### 2.1. Material and methods

Commercial PP, T300 (Mn = 8 × 10^4^, Mw = 3.3 × 10^5^) was provided by Shanghai Petrochemical LCD of P.R. China, with MFR (melt flow rate) of 3.5 g/10 min at 190°C. Pure PA6 pellets (YH-800) were purchased from Yueyang Petrochemical Co. Ltd. These had a density of 1.14 g cm^−3^, a melting point of 215–220°C and a MFR of 28 g/10 min at 230°C with 2.16 kg load. CB (VXC-72) was supplied by Cabot China Ltd, with a nitrogen absorption specific surface area of 254 m^2^ g^−1^ measured by the BET method. The particle size of CB was around 30 nm and the density at room temperature was 0.256 g cm^−3^.

### 2.2. Sample preparation

All materials were dried at 100°C in vacuum for 10 h before use. The PP/PA6 blends filled with different CB concentrations from 0 to 12% were prepared by melt mixing in an internal mixer (SU-70C, Suyan Science and Technology Co. Ltd, China). Samples were mixed for 8 min at 190°C and 30 r.p.m. (only with the PP matrix) or at 230°C and 60 r.p.m. (both with PP and PA6 matrices). The mixtures obtained were compression-moulded into samples of 1 mm thick at 195 or 235°C, respectively. They were first preheated at 195°C or 235°C for 5 min, and then pressed at 100 bar for 4 min before being cooled to room temperature for 3 min.

### 2.3. Characterization

#### 2.3.1. Rheological measurements

Dynamic rheology measurements were performed using an advance rheometric expanded system (ARES, Rheometrics, USA) with a parallel plate gripper of 25 mm in diameter. Dynamic frequency sweeping from 500 to 0.1 rad s^−1^ was performed at 245°C under dry nitrogen atmosphere. The strain used was 1% which ensured to be in the linear regime. Elastic modulus (*G*′), viscous modulus (*G*′′), and complex viscosity η* were measured in the frequency sweep experiments.

#### 2.3.2. Thermal properties and crystallinity

The crystallization behaviour of the samples was studied under nitrogen atmosphere by differential scanning calorimetry (model DSC-Q200, TA Instruments, USA), using 5–8 mg of sample sealed into aluminium pans. At first, the samples were heated from ambient temperature to 250°C with a heating rate of 20°C min^−1^ and held at 250°C for 5 min to melt and erase any thermal history, then cooled down to 40°C with a cooling rate of 10°C min^−1^. The second heating was similar to the first one with a heating rate of 10°C min^−1^. The crystallization and melting temperatures for each component were obtained by the cooling and second-heating curves, respectively. The crystallinity (*χ*_c_) of the composites was calculated with the following equation:

2.1 $$\chi_{c}=\frac{\Delta H_{m}}{\Delta H_{m}^{0}\times w}\times 100\%,$$

where Δ*H*_m_ is the enthalpy of fusion for PA6 or PP in the blends, $\Delta H_{m}^{0}$ represents the enthalpy of fusion for 100% crystalline PA6 or PP and *w* means the weight fraction of specific polymer component in the blending composite. The enthalpies of fusion for 100% crystalline PA6 and PP are 230 J g^−1^ [21] and 170 J g^−1^ [22], respectively.

#### 2.3.3. Morphology and microstructure

The phase structure of the blends was examined by scanning electron microscopy (SEM; S-4800, Hitachi, Japan). The moulded specimens were prepared after the fracture of the composite sheets in liquid nitrogen and sprayed deposition of a thin gold layer onto the surface. To obtain a better contrast, the PA6 phase of some samples was etched away with formic acid at room temperature for 1 h, after which the etched specimens were washed with ethanol.

#### 2.3.4. Electrical conductivity

The surface resistivity (*ρ*_s_) was measured on compression-moulded plaques with dimensions of 80 × 80 × 1 mm^3^ using a ZC-90 megohmmeter under the condition of 25°C and 65% relative humidity. The ZC-90 megohmmeter was preheated for half an hour while the samples and electrodes were cleaned with ethanol before measurements. Electrical conductivity of each sample was measured five times at an applied voltage of 500 V, and the results were reported as average values.

## 3. Results and discussion

### 3.1. Theoretical calculation of the location of carbon black in the polypropylene/polyamide 6 blends

In general, the location of CB in an immiscible PP/PA6 blend is predicted by calculating the wetting coefficient *ω*_a_ according to Young's equation [23]:

3.1 $$\omega_{a}=\frac{\gamma_{\mathrm{CB}\text{–}\mathrm{PP}}-\gamma_{\mathrm{CB}\text{–}\mathrm{PA}6}}{\gamma_{\mathrm{PP}-\mathrm{PA}6}},$$

where *γ*_CB–PP_, *γ*_CB–PA6_ and *γ*_PP–PA6_ are the interfacial tensions between CB and PP, between CB and PA6, and between PP and PA6, respectively. If *ω*_a_ is higher than 1, the CB particles will be located in PA6; if it is between −1 and 1, the CB particles will be preferentially distributed at the interface between the two polymers; if *ω*_a_ is lower than −1, the CB particles will prefer to distribute in PP.

The interfacial tension can be calculated using the harmonic-mean equation:

3.2 $$\gamma_{\mathrm{AB}}=\gamma_{A}+\gamma_{B}-4\;\left(\frac{\gamma_{A}^{d}\gamma_{B}^{d}}{\gamma_{A}^{d}+\gamma_{B}^{d}}+\frac{\gamma_{A}^{p}\gamma_{B}^{p}}{\gamma_{A}^{p}+\gamma_{B}^{p}}\right)$$

and the geometric-mean equation:

3.3 $$\gamma_{\mathrm{AB}}=\gamma_{A}+\gamma_{B}-2\sqrt{\gamma_{A}^{d}\gamma_{B}^{d}}-2\sqrt{\gamma_{A}^{p}\gamma_{B}^{p}},$$

where *γ*_*i*_ is the surface tension of component *i*, and $\gamma_{i}^{d}$ and $\gamma_{i}^{p}$ are, respectively, the dispersive and polar components.

However, the surface tensions of PP and PA6 at the compounding temperature (230°C) are required. Values of surface tension at the compounding temperature are calculated as follows:

$$-\frac{d\gamma }{dT}=0.06\;\text{and}\;-\frac{dx^{P}}{\text{d}T}=\frac{\text{d}(\gamma^{P}/\gamma )}{\text{d}T}=0,$$

where (−d*γ*/d*T*) is the variation rate of surface tensions with temperature and *x*^p^ = *γ*^p^/*γ* is the polarity of polymer.

Table 1 lists the surface tensions of PP, PA6 and CB at 230°C which are available in the literature [23,24]. The interfacial tensions between the different blend components can be calculated based on equations (3.2) and (3.3), and the results are shown in table 2. From the values of wetting coefficient calculated based on equations (3.2) and (3.3) (3.48 and 4.01, respectively), this means that the CB particles should selectively distribute in the dispersed phase, i.e. PA6.

**Table 1.** Surface tension of PP, PA6 and CB at 230°C.

polymer	*γ*(mN m^−1^)	*γ*^d^(mN m^−1^)	*γ*^p^(mN m^−1^)
PP	17.62	17.25	0.37
PA6	37.19	35.00	2.19
CB	85.50	82.36	3.14

**Table 2.** Interfacial tensions and wetting coefficient at 230°C.

	*γ*_CB–PP_ (mN m^−1^)	*γ*_CB–PA6_ (mN m^−1^)	*γ*_PP–PA6_ (mN m^−1^)	*ω*_a_
harmonic-mean equation (3.2)	19.28	44.75	7.32	3.48
geometric-mean equation (3.3)	10.07	25.58	3.87	4.01

**Figure 1.**
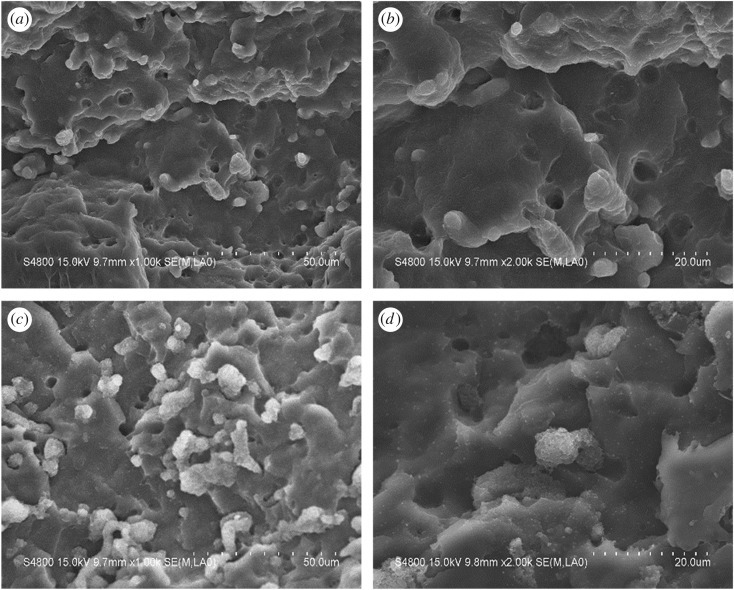
SEM micrographs of freeze-fractured surfaces of (*a*,*b*) PP/PA6/CB (80/20/8) and (*c*,*d*) PP/PA6/CB (80/20/8, etched) at two magnifications.

To verify the true location of CB, the composite samples were examined by SEM after etching away the PA6 phase with formic acid. Here, it was noted that, during the etching process, black precipitate appeared in the formic acid and the solution became darker with the increase of the PA6 content in the composites, giving evidence of CB's presence in the PA6 phase. Figure 1 shows SEM images of surfaces of the PP/PA6/CB (80/20/8) before and after etching. In the PP/PA6/CB (80/20/8) blend, PA6 disperses in the continuous PP matrix and forms spherical particles of 20–60 µm in diameter, as shown in figure 1*a,b*. The two-phase structure in the composite system is obvious, and the interfacial bonding between PP and PA6 is weak, leading to quenching at the interface between PP and PA6. Some PA6 particles fall off from the PP matrix at the cross section, indicating the poor compatibility between PP and PA6. Figure 1*c,d* clearly shows the exposed CB particles after etching, indicating that the CB is preferentially distributed in the PA6 phase.

### 3.2. Electrical conductivity of the polypropylene/polyamide 6/carbon black composites

**Figure 2.**
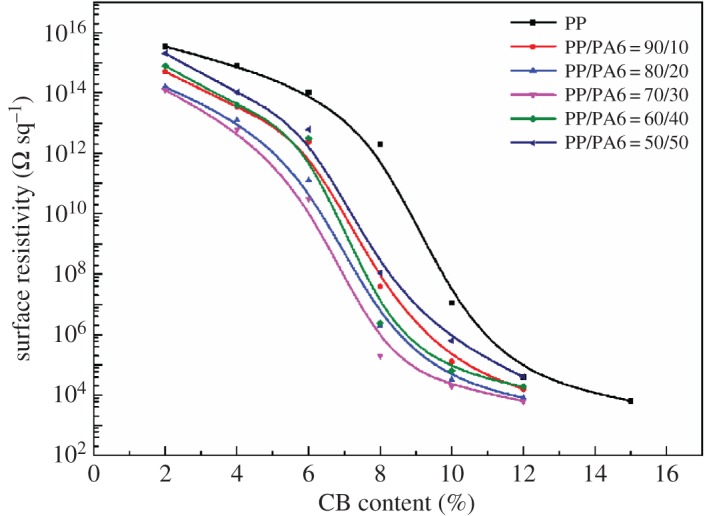
Surface resistivity versus CB mass fraction (*w*) of the PP/CB and PP/PA6/CB composites.

Figure 2 shows the percolation behaviours of the ternary and binary composites. For the PP/CB composite, surface resistivity *ρ*_s_ changes slightly at low CB content (*w *< 0.06), and CB particles or their aggregates are dispersed randomly in the matrix which cannot form a conductive path throughout the entire material. The conductive process of the composite material mainly depends on the polymer matrix, showing insulator characteristic. With the increase of the CB content, CB particles begin to contact with each other to form continuous conductive path at the range of 0.06 to 0.12, leading to a sharp drop of *ρ*_s_, resulting in the transition of material from insulator to semiconductor. After the CB content exceeds the percolation threshold, the influence of *w* on *ρ*_s_ is reduced and the decreasing trend of the curve is slowed down due to the formation of the conductive network.

The measured surface resistivity of the nanocomposites containing different amounts of CB is given in table 3. In all samples, electrical conductivity increased with higher amounts of CB. Compared with CB-filled single-matrix system, the percolation of the CB-filled PP/PA6 system is greatly reduced. When *w* is less than 0.06, the surface resistivity of PP/PA6 system decreases by 2–4 orders of magnitude. It can be seen that adding the third component PA6 to the PP/CB system can significantly reduce the resistivity of the composite with low CB content. For the same CB content, the surface resistivity of the PP/PA6/CB composite is smaller than that of the PP/CB composite system, resulting in the better conductivity of the composite.

**Table 3.** Surface resistivity (Ω sq^−1^) of different nanocomposites.

CB content	surface resistivity (Ω sq^−1^)					
(wt%)	PP	PP/PA6 (90/10)	PP/PA6 (80/20)	PP/PA6(70/30)	PP/PA6 (60/40)	PP/PA6 (50/50)
6	1.04 × 10^14^	2.40 × 10^12^	1.25 × 10^11^	3.16 × 10^10^	3.17 × 10^12^	6.31 × 10^12^
8	2.00 × 10^12^	3.98 × 10^7^	2.00 × 10^6^	2.01 × 10^5^	2.40 × 10^6^	1.10 × 10^8^
12	4.00 × 10^4^	1.58 × 10^4^	7.94 × 10^3^	6.30 × 10^3^	1.91 × 10^4^	3.98 × 10^4^

The percolation phenomenon of CB-filled polymer blend is not the same as that of a single-polymer matrix. For the polymer blend, in general, a low weight fraction (minority) polymer blend component often forms a dispersed phase with a sea–island microstructure. At a critical concentration, the microstructure inverts from a sea–island microstructure to co-continuous network as the weight fraction of the minor phase increases. The requirement for electrical conductivity is the formation of a continuous conductive filler pathway and when the conductive filler partitions to one phase, that phase becomes conductive when the percolation threshold is exceeded. Owing to the local enrichment of conductive particles caused by uneven distribution of CB in two phases, fewer CB particles can form the conductive network; as a consequence, the percolation threshold of CB particles is reduced greatly.

**Figure 3.**
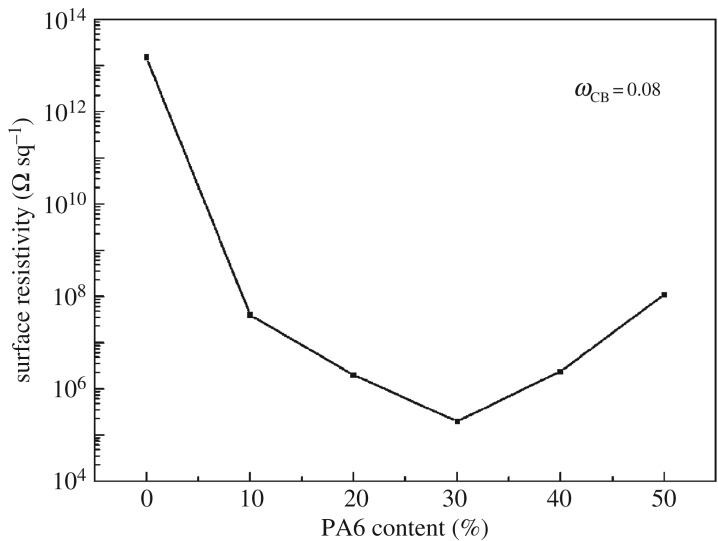
Surface resistivity of the PP/PA6/CB composites as a function of PA6 content (8 wt% of CB).

Figure 3 shows the surface resistivity of PP/PA6/CB composites with 8 wt% of CB as a function of PA6 content. With a PA6 content of 0 wt%, i.e. the PP/CB composite, the samples were not electrically conductive (1.49 × 10^13^ Ω sq^−1^). The addition of 10 wt% of PA6, which is an insulating material, resulted in an increase in electrical conductivity by more than six orders of magnitude. With the increase of the content of PA6, the conductivity increased slightly further. The change can be explained by the preferential location of CB in PA6 phase. On addition of PA6, the CB concentration in the PA6 phase rises and reaches the percolation threshold, leading to a steep conductivity increase. When the PA6 content exceeds 30%, the concentration of CB in PA6 is reduced and it is difficult for CB particles to form a continuous conductive network, resulting in a decrease of resistivity. It can be seen that the introduction of PA6 into PP could remarkably decrease the resistivity of composites, and a minimum surface resistivity appeared when the weight ratio of PP to PA6 is 70/30, reaching a value of 2.01 × 10^5^ Ω sq^−1^. It can be concluded that PA6 formed a relatively perfect continuous conductive path in the system.

### 3.3. Morphologies of the polypropylene/polyamide 6/carbon black composites

The location of the conductive filler and the phase morphology of the composites could considerably affect the electrical properties of the polymer blends, and it is clear that the phase morphology can be changed by adjusting the CB concentration and the PP/PA6 ratio.

**Figure 4.**
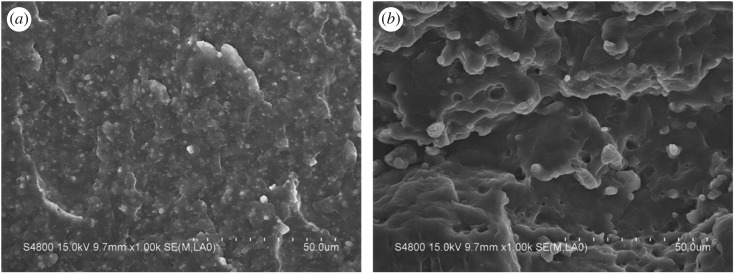
SEM micrographs of freeze-fractured surfaces of PP/PA6 blends with 8 wt% of CB of composition of (*a*) 90/10, (*b*) 80/20.

**Figure 5.**
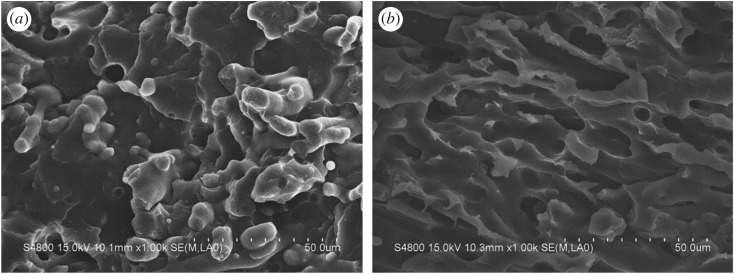
SEM micrographs of freeze-fractured surfaces of PP/PA6/CB (70/30/8).

**Figure 6.**
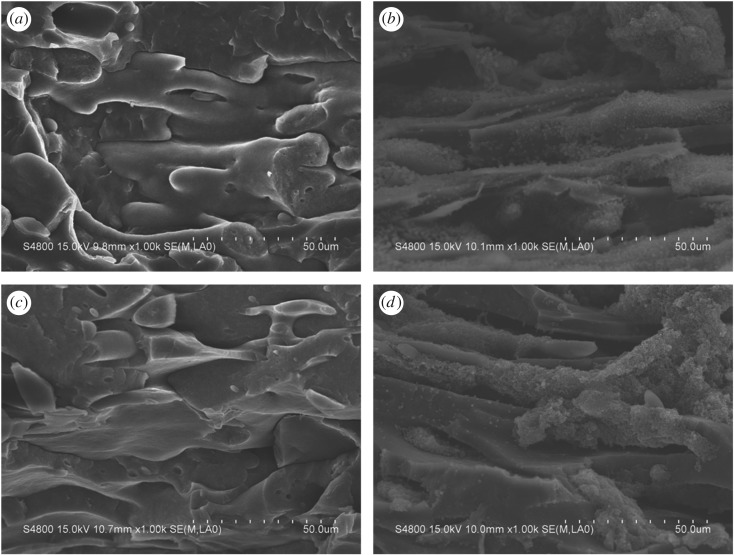
SEM micrographs of freeze-fractured surfaces of PP/PA6 blends with 8 wt% of CB of composition of (*a*) 60/40, (*b*) 60/40 etched, (*c*) 50/50, (*d*) 50/50 etched.

Figures 4–6 show SEM images of surfaces of the PP/PA6 blends filled with 8 wt% CB at different PA6 contents. For the PP/PA6 (90/10) and PP/PA6 (80/20) blends filled with 8 wt% CB (figure 4), the PA6 was a discrete minor phase dispersed in a continuous PP phase matrix, i.e. a typical sea–island morphology. The space between PA6 phases was too large to form a continuous structure, so the resistivity of the composite was relatively large.

For the composites of PP/PA6 (70/30), the phase structure of the system changed obviously (figure 5). As PP and PA6 are immiscible and incompatible, the low interfacial adhesion leads to the extraction of PA6 phase during the process of fracture in liquid nitrogen. The morphologies of the composites formed two parts: (a) microfibrillar structure and (b) micropore structure. PA6 phase formed a long and dense fibrous structure in the PP matrix, and the fibres overlapped with each other, forming a continuous structure, which can significantly improve the conductivity of the composite material.

The structure of PA6 phase in the composites of PP/PA6 (60/40) and PP/PA6 (50/50) (figure 6) also showed a fibrous structure, similar to that of PP/PA6 (70/30). However, the fibres were relatively short and the distribution is not that regular, leading to the poor continuity of PA6 phase, so that the conductivity of the material is deteriorated, and the surface resistivity is slightly higher than the former.

### 3.4. Linear rheology

**Figure 7.**
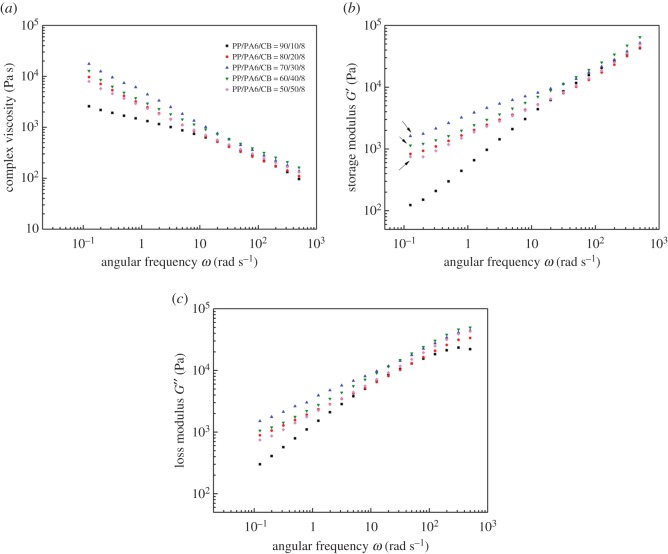
Frequency-dependent viscoelastic properties of PP/PA6/CB composites: (*a*) complex viscosity *η*^∗^, (*b*) storage modulus *G*′ and (*c*) loss modulus *G*′′.

Research studies of conductive composite materials have shown that the rheological flow threshold of the composites is consistent with the conductive seepage threshold, and the dynamic rheological behaviour is related to the conductivity. Linear rheology is effective for assessing the state of dispersion of fillers in polymer melts [25,26]. Figure 7 illustrates the complex viscosity, storage modulus and loss modulus as a function of angular frequency for the ternary composites with 8 wt% of CB. As observed in the complex viscosity curves, samples exhibit shear-thinning behaviour with increasing frequencies, indicating a pseudoplastic nature. When the content of PA6 is low, the spacing between CB particles is too large to form the network structure. What plays a dominant role in viscosity is the entanglement characteristics between polymer matrixes. Viscosity curve of the PP/PA6 (90/10) shows a plateau at low frequencies. By introducing and increasing the amount of PA6 in the blend, this plateau vanishes, the slopes of the curves increase, and a viscosity upturn is observed. This behaviour is usually attributed to the existence of a percolated network in the polymer matrix. At this point, the network structure comes to play a dominant role. It is worth noting that the 70/30 system has a higher viscosity value than the 60/40 and 50/50 systems. Because of the selective dispersion of CB particles in the PA6 phase, when *w*_CB_ is kept the same (8 wt% of the whole system), as the PA6 content in these composites is increased, the relative content of CB in PA6 phase decreases. This leads to a decrease in the viscosity of the PA6 phase and the whole system.

Moreover, the storage modulus increases at higher CB contents, and slopes of the curves decrease. This phenomenon can also be explained by the existence of a mechanically percolated network because of the interactions between PP matrix and PA6. When PA6 content is lower than 20 wt%, the interparticle distance is too large to form the filler-network structure. The storage modulus of PP/PA6/CB with 20 wt% of PA6 content, in the low frequency region, is much higher than that of the composite with 10 wt% of PA6 content due to the decrease of interparticle distance. After adding 30 wt% PA6, PA6 formed continuous structure in the system, and the conductivity of the system was improved obviously. It can be concluded that the CB in the PA6 and PA6 in the composite system both formed a better continuous conductive network structure; therefore, *G*′ value is larger in the low frequency region. Also, a small platform (as indicated by the arrow) is observed at low frequency region of *G*′. Moreover, the platform becomes more and more obvious as the PA6 content continues to increase. Since this platform at low frequency region has been observed in many studies due to the network structure of the fillers [27–29], we can confirm that a perfect continuous structure is indeed present in blends with higher PA6 content (40 and 50%). The storage modulus of all samples in the high frequency region is almost the same, owing to the mechanical saturation. This result further confirms the electrical performance result and SEM result obtained in the previous section.

However, even at higher filler contents, slopes of the *G*′′ curves of blend nanocomposites do not change too much, suggesting that a full volume-spanning network has not been formed in the samples. This is probably due to the large localization of CB in the PA6 phase.

### 3.5. Crystallization and melting behaviours

**Figure 8.**
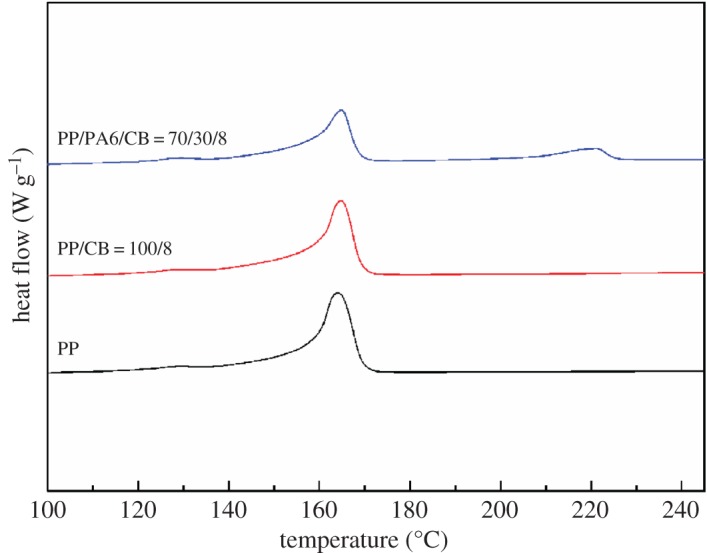
DSC heating thermograms of PP/PA6 blend with 8 wt% CB.

**Figure 9.**
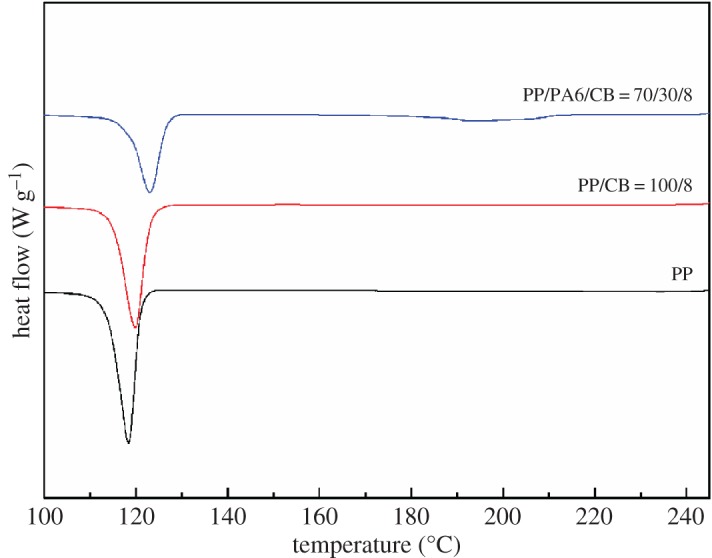
DSC cooling thermograms of PP/PA6 blend with 8 wt% CB.

Figures 8 and 9 reflect the effect of PA6 on the crystallization behaviour of the system. For clarity, all DSC curves shown here are shifted vertically. Both PA6 and PP melting peaks are detected at their expected positions in the melting curves, typical of immiscible blends. The crystallization peak temperature (*T*_c_), melting peak temperature (*T*_m_) and crystallinity (*χ*_c_) of PP/PA6/CB specimen with 8 wt% of CB are shown in table 4.

**Table 4.** DSC data of PP/CB and PP/PA6/CB composites.

	PP			PA6		
samples	*T*_c_ (°C)	*T*_m_ (°C)	*χ*_c_	*T*_c_ (°C)	*T*_m_ (°C)	*χ*_c_
neat PP	118.4	164.1	52.4	—	—	—
neat PA6	—	—	—	184.6	219.5	19.8
PP/CB (100/8)	119.9	164.7	52.1	—	—	—
PP/PA6/CB (70/30/8)	123.1	164.8	52.0	193.8	221.1	22.9

From figure 8, it can be seen that the PP/PA6/CB (70/30/8) sample exhibits a strong main melting peak for PP (approx. 165°C) and a weak melting shoulder for the PA6 matrix (approx. 220°C). It is clear from table 4 that the crystallinity of the PA6 component is higher than that of the neat PA6 while the crystallinity of PP changes less. The increase of crystallinity for PA6 can be explained in that CB particles are mainly dispersed in PA6, which has a more pronounced nucleation effect on PA6 crystallization, leading to the reduction in wafer thickness and the increase in crystallinity of PA6 phase. The increase in the crystallinity of the PA6 phase could have contributed to the sharp increase in the electrical conductivity. As the CB particles will populate in the amorphous regions, higher crystallinity can further increase the effective concentration of CB in the PA6 phase. Higher crystallinity of the polymer may cause the higher conductivity, because the conductive filler is preferentially dispersed in the amorphous state, resulting in a relatively increased concentration of the conductive filler.

Unlike the melting behaviour of the composites, the crystallization process is greatly affected by the incorporation of PP and CB into the PA6 matrix. Observing the DSC cooling curves (figure 9) and table 4, it is clearly seen that the crystallization temperatures for both components of the immiscible composites greatly shift to higher temperatures when compared with those of neat PA6 and PP, respectively. This is possibly because at the crystallization temperature of each phase, the surface of the other blend component (solid or melt) can act as heterogeneous nucleation sites, increasing the nucleation rate.

## 4. Conclusion

In this work, rheology, morphology and electrical conductivity of CB-filled immiscible PP/PA6 blends were investigated as a function of CB content and PP/PA6 blending ratio. Based on the results, the following conclusions can be drawn:

1. The SEM images and electrical conductivity measurements confirm that the CB is preferentially located in the PA6 phase. In the case of the PP/PA6 blends with 8 wt% of CB, the addition of PA6 promotes the morphological change from a typical sea–island morphology to a co-continuous morphology.
2. The selective localization of the CB particles increases their effective concentration in the PA6 phase. Therefore, denser networks can be formed and higher electrical conductivity can be obtained in comparison with neat PP at the same filler loadings. With 8 wt% of CB, the highest electrical conductivity is found when the PP and PA6 phases form a co-continuous structure at a PP/PA6 ratio of 70/30, reaching a value of 2.01 × 10^5^ Ω sq^−1^.
3. Although the rheological percolated network structure cannot be deduced apparently in this work, the variations of both complex viscosity and the storage modulus hint at the changes of the microstructures of the PA6 in the composites.
4. The crystallization behaviours further prove that the crystallite growth of PA6 phase is restricted by the CB to a certain extent. The higher crystallinity of the polymer may cause higher conductivity, due to the fact that the conductive filler is preferentially dispersed in the amorphous state, resulting in a relatively increased concentration of the conductive filler.

## Data accessibility

All necessary data are provided in Results and discussion section in the main text.
